# Research on Educational Robot System Based on Vision Processing

**DOI:** 10.3390/s23021038

**Published:** 2023-01-16

**Authors:** Jianwei Zhao, Yutian Gu, Qifeng Hou, Zhiwei Zhang

**Affiliations:** 1 School of Mechanical Electronic and Information Engineering, China University of Mining and Technology, Beijing 100089, China; 2 Institute of Artificial Intelligence, University of Science and Technology Beijing, Beijing 100083, China

**Keywords:** educational robot, three closed-loop PID control, multi-sensor fusion, cascade classifier

## Abstract

Aimed at the poor recognition effect of current educational robots on objects with complex shapes and colors and the single design of related experiments, this paper proposes a robot teaching instrument. The robot adopts a servo motor with an encoder, a drive, and a variety of sensors to realize a motor current loop, speed loop, position loop, and closed-loop control functions. Three experimental schemes were designed: a PID adjustment experiment, a robot obstacle avoidance and object-grasping program writing experiment, and a complex object recognition experiment based on cascade classifiers. The robot is conducive to improving students’ self-initiative ability, deepening their understanding of PID closed-loop control, multi-sensor fusion, and deep learning knowledge. It can improve students’ programming ability, enabling them to effectively combine theory and practice, as well as to comprehensively apply professional knowledge.

## 1. Introduction

Robot-based education indicates a robot specially developed in the field of education for the purpose of cultivating students’ analytical, creative, and practical ability. It is characterized by educational applicability, openness, expansibility, and friendly human–computer interaction. It is used to motivate students’ learning interests, cultivate their comprehensive ability, and improve the learning experience of students.

At present, the problems in the application of educational robots in China include the following: (1) There is more theoretical knowledge and less practical teaching. (2) The focus is on software simulation and program writing, whereas hardware debugging knowledge is less developed. (3) There is only a relatively simple introduction to cutting-edge knowledge and a lack of more in-depth teaching. In response to these problems, Lv Hai [1] proposed an educational robot experiment system based on MATLAB, which expanded the module library and experimental case library of the test system. Wang Pan [2] proposed a children’s education robot based on multi-sensor fusion with an improved RBPF-SLAM algorithm to further improve the accuracy and performance of the algorithm. Yujia W et al. [3] proposed an embedded educational robot based on MCU+FPGA, which has further improved the integrity of software and hardware systems. Yujia L et al. [4] proposed a gesture recognition educational robot based on an inertial sensor, which has better openness and scalability. Combined with the above research results, considering the problems of related educational robots, a wheeled educational robot based on multi-sensor fusion and deep learning is proposed, and three experimental courses from shallow to deep are designed to solve some problems in the use of educational robots in educational teaching activities.

## 2. Structure Design of Educational Robot

### 2.1. Robot Mathematical Model Analysis

The educational robot designed in this paper is a wheeled mobile robot with four symmetrical wheels, which are driven independently. It is mechanically simple and highly flexible. The automobile body is not provided with a steering device and relies on differential movements of the left and right wheels to realize different radius steering abilities and even zero radius steering. However, because of its nonlinear, time-varying, multi-variable, strong coupling and other characteristics, its movement has greater uncertainty than the movement of a body with a steering device, so it cannot use a simple kinematic model to represent its movement characteristics, and it is necessary to build kinematic models [5].

**Figure 1:**
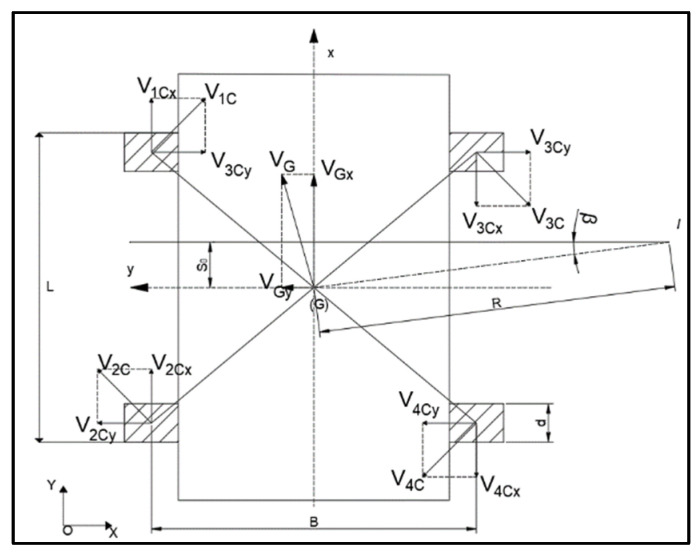
Mathematical model.

As shown in Figure 1, it is considered that the car body rotates around the center of the moment I with a radius R and rotates at the speed V_G_. The center of mass of the robot is G, the ground coordinate is XOY, and the body coordinate system is x-y connected with the center of mass. B is the track width, L is the wheelbase, and d is the longitudinal length of the wheel in contact with the ground. The following mathematical model is established [6].

In the fuselage coordinate system:

$$\left\{\begin{array}{c} v_{x}=v\cos\alpha \\ v_{y}=v\sin\alpha \end{array}\right.$$

In the ground coordinate system, the airframe velocity can be decomposed as:

$$\left\{\begin{array}{c} \dot{X}=v_{x}\cos\theta -v_{y}\sin\theta \\ \dot{Y}=v_{x}\sin\theta +v_{y}\cos\theta \end{array}\right.$$

In the process of motion, the relative sliding between the mobile robot and the ground is inevitable. In this paper, the sliding rate is used to describe the sliding situation of the wheel:

$$s_{i}=\frac{w_{i}r-v_{ix}}{w_{i}r}\times 100\%$$

$w_{i}$—speed of wheel I;

$v_{ix}$—x-direction component velocity of wheel center of wheel I.

1. When $s_{i}>0$ (the linear velocity of the wheel is greater than the velocity of the wheel center), the friction force between the wheel and the ground is the driving force, and the slip occurs at this time [7].
2. When $s_{i}<0$ (the linear speed of the wheel is less than the speed of the wheel center), the friction force between the wheel and the ground is the braking force, and slip occurs at this time.
3. When $s_{i}=0$ (the linear velocity of the wheel is equal to the velocity of the wheel center), the robot is in a fully rolling state.

Define the side near the center of rotation as inboard and the other side as outboard.

When the robot steers, the inside wheel slips, the outside wheel turns, and the instantaneous center of the contact point between the wheels on both sides and the ground is equal to the y coordinate of the rotation center [8].

During the process of rotation, the longitudinal speed of the wheel center on the same side is equal, that is:

$$w_{1}r\left(1-s_{1}\right)=w_{2}r\left(1-s_{2}\right)\;w_{3}r\left(1-s_{3}\right)=w_{4}r\left(1-s_{4}\right)$$

Because the wheel on the same side rotates at the same speed, the following is true:

$$s_{1}=s_{2}=s_{l},\;s_{3}=s_{4}=s_{r}$$

The relationship between the linear velocity, the attitude angular velocity, and the rotation speed of the left and right wheels is:

$$\left\{\begin{array}{c} v=\frac{w_{l}r+w_{r}r}{2}\\ \dot{\varphi }=\frac{w_{r}r-w_{l}r}{b} \end{array}\right.$$

Because of the slip, the linear velocity is represented by the wheel center longitudinal velocity, and the lateral velocity may be represented by the slip angle.

As such, the following can be obtained:

$$\left[\begin{array}{c} v_{x}\\ v_{y}\\ \dot{\varphi } \end{array}\right]=\frac{r}{2}\left[\begin{array}{cc} 1-s_{l} & 1-s_{r}\\ \tan\alpha \left(1-s_{l}\right) & \tan\alpha \left(1-s_{r}\right)\\ -\frac{2\left(1-s_{l}\right)}{b} & \frac{2\left(1-s_{r}\right)}{b} \end{array}\right]\left[\begin{array}{c} w_{l}\\ w_{r} \end{array}\right]$$

The kinematics equation of the mobile robot in the ground coordinate system XOY can be obtained through coordinate system transformation as follows (Table 1):

$$\left[\begin{array}{c} \dot{X}\\ \dot{Y}\\ \dot{\theta } \end{array}\right]=\left[\begin{array}{ccc} \cos\theta & \sin\theta & 0\\ -\sin\theta & \cos\theta & 0\\ 0 & 0 & 1 \end{array}\right]\left[\begin{array}{c} v_{x}\\ v_{y}\\ \dot{\varphi } \end{array}\right]$$

### 2.2. Robot Entity Construction

#### 2.2.1. Overall Structure of Vehicle Body

**Figure 2:**
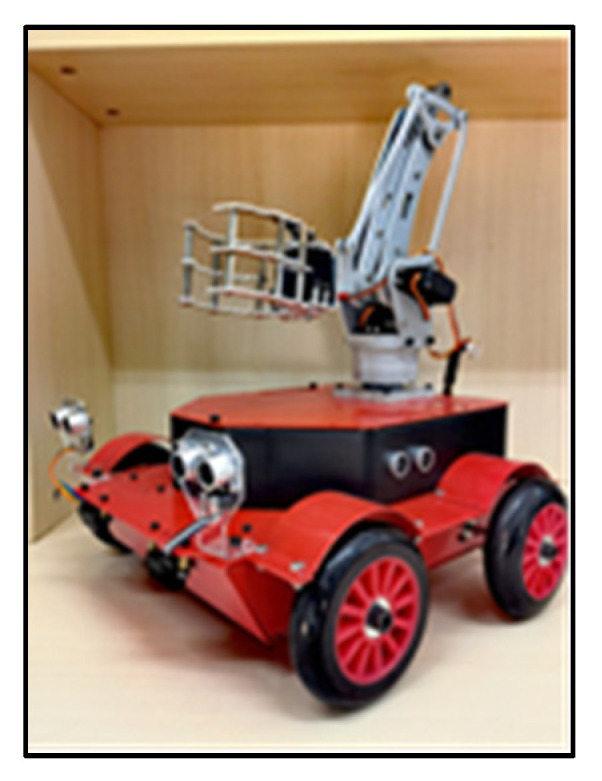
Overall picture of the car body.

As shown in Figure 2, the vehicle body is divided into an upper layer and a lower layer. The lower layer of the vehicle body is mainly responsible for carrying the motor and the drive board, which is responsible for the motion control of the vehicle body. 

The second layer of the car body is equipped with ultrasonic and infrared modules to measure the distance of objects around the body and target objects. The mechanical arm is installed on the top of the body and is responsible for snatching the target objects within 20 cm around.

#### 2.2.2. Chassis Structure

**Figure 3:**
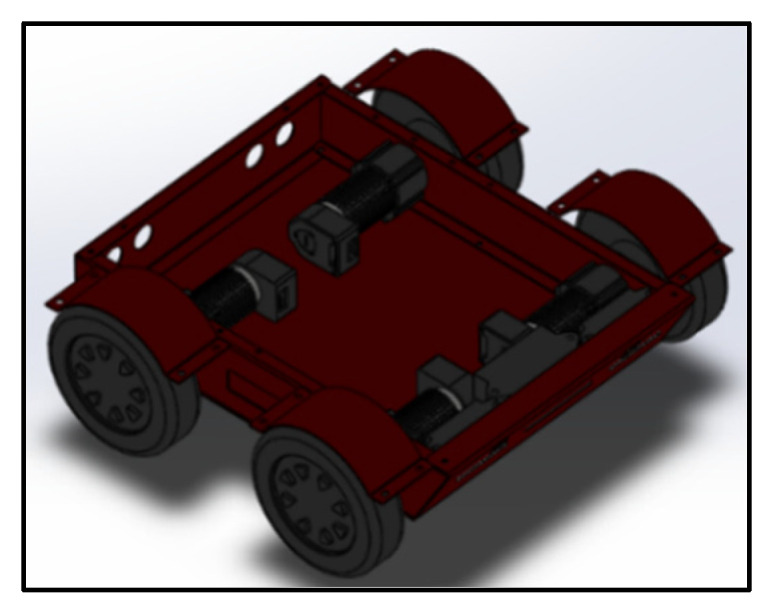
Chassis structure diagram.

The chassis structure of the trolley is shown in Figure 3. It is mainly used to carry the driving structure of the vehicle body, and four brushless motors and driving boards are installed at the bottom. Two ultrasonic detection sensors through holes are reserved at the rear side of the vehicle body to carry the ultrasonic sensors, the front end of the vehicle body is designed to have a slope with a certain angle, and an opening is reserved at the side position to facilitate the connection and debugging of data lines. The whole chassis is made of aluminum with a thickness of two millimeters, which is mainly responsible for the load bearing of the car body [9].

#### 2.2.3. Sensors

1. Ultrasonic transducers

**Figure 4:**
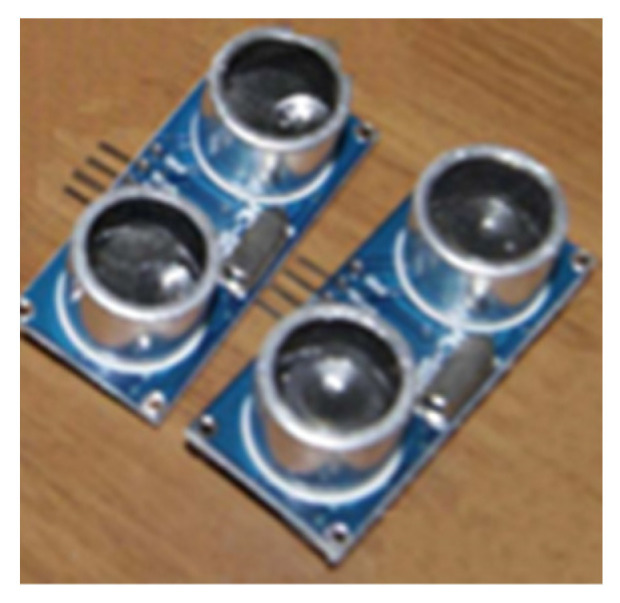
Ultrasonic transducers.

At present, there are many types of ultrasonic sensors on the market. We chose the HC-SR04 ultrasonic module shown in Figure 4, which uses a voltage of DC 3.3–5 V, has a smaller operating current (3 mA) under the same range, a detection distance of 2–450 cm, and an accuracy of up to 3 mm.

2. Infrared sensor

**Figure 5:**
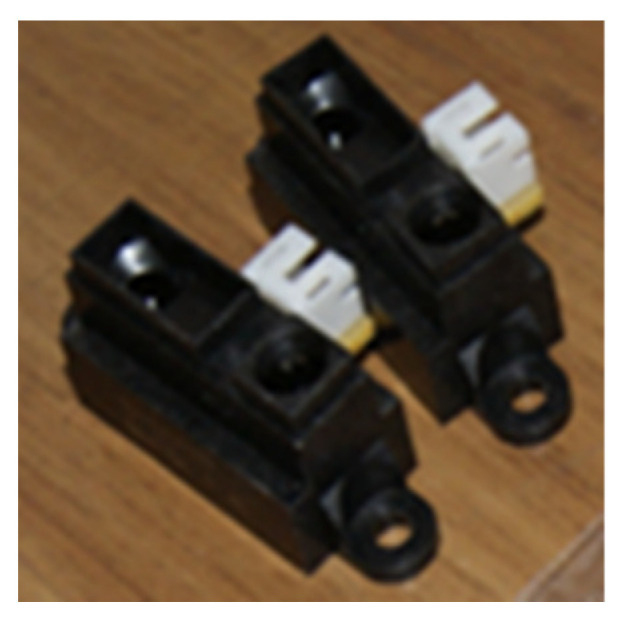
Sensor element.

This robot uses the GP2Y0A2YK0F infrared sensor shown in Figure 5. The modified infrared adopts the triangulation method, and the material, ambient temperature, and measurement time of the measured object will not affect the measurement accuracy of the sensor. The sensor output voltage value corresponds to the detected distance.

#### 2.2.4. Motor

**Figure 6:**
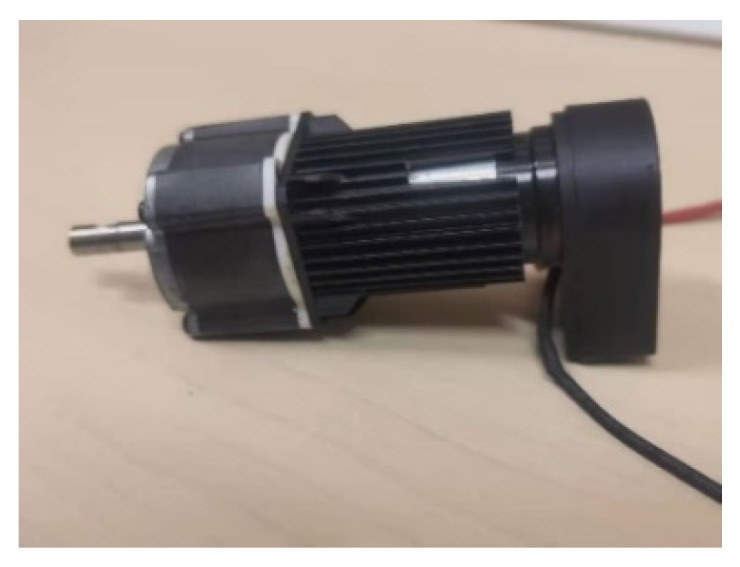
Motor.

Figure 6 shows the motor selected for this robot. The motor used to drive the robot is the Von Haber hollow cup gear motor. The hollow cup motor has three characteristics: a high energy conversion utilization rate, fast speed adjustment, and small rotation fluctuation. An encoder is installed on the motor to feed back the operation information of the motor. Four motors are installed on the robot chassis to drive and control the vehicle body [10].

#### 2.2.5. Motor Drive Board

**Figure 7:**
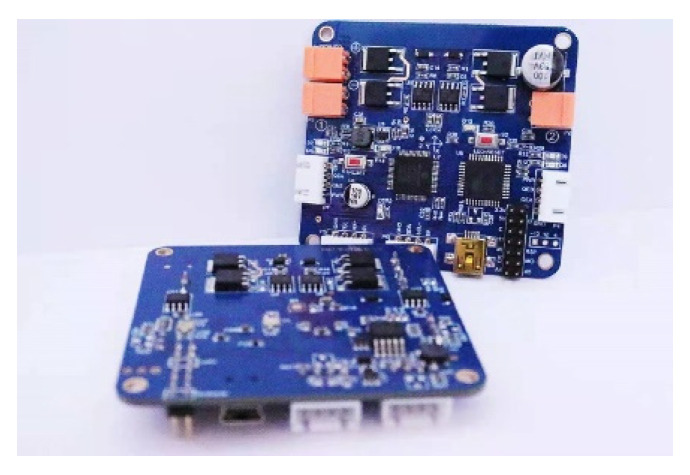
Motor drive board.

The motor drive board is the DC servo PID motor drive board shown in Figure 7. The drive has a built-in 32,768 bit resolution absolute magnetic encoder. It has two motor interfaces, which each output 5 A for motor control, and CANOPEN and RS485 interfaces, which can read the current speed and position in real time for easy PID control. By controlling this drive board, the motor can be controlled in a closed loop.

### 2.3. Circuit Connection

**Figure 8:**
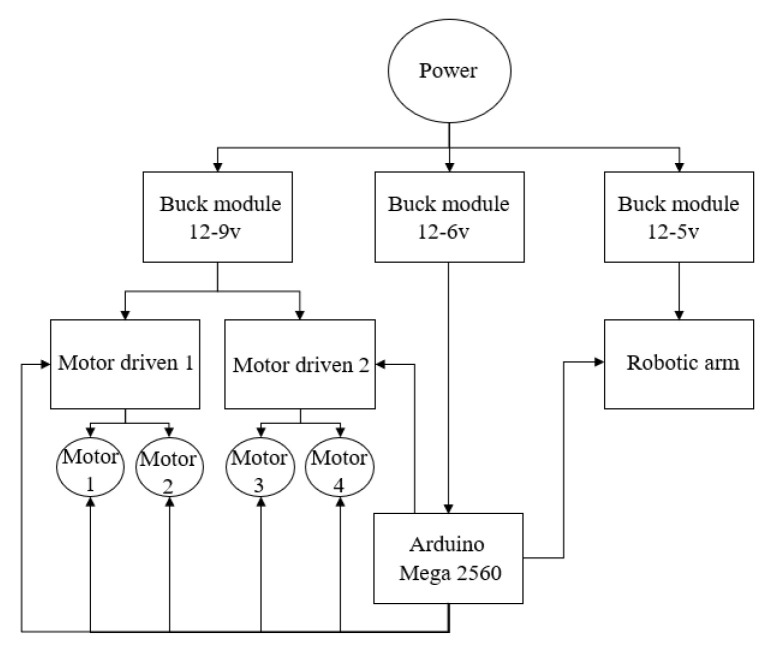
Circuit connection diagram.

As shown in Figure 8, the trolley power supply is powered by a 12 V DC battery, and after the power supply battery is powered, the 12 V voltage is first reduced to 6 V voltage through the step-down module. The 5 A current provides power to the Arduino drive board, and at the same time, through the second step-down module, the power supply voltage is reduced to 5 V, the three servos on the robotic arm are separately powered, and the third step-down module drops the voltage to 9 V and supplies power to two motor drive boards at the same time. Each motor drive board controls two motors at the same time [11,12].

In terms of control line, the whole vehicle is controlled by the Arduino mage2560 drive board, which is connected to the motor drive board through the line, and the speed and steering of the four motors are controlled by the control motor drive board. The robotic arm is controlled directly by the Arduino board.

## 3. Computer-Based Identification System

### 3.1. System Design Idea

**Figure 9:**
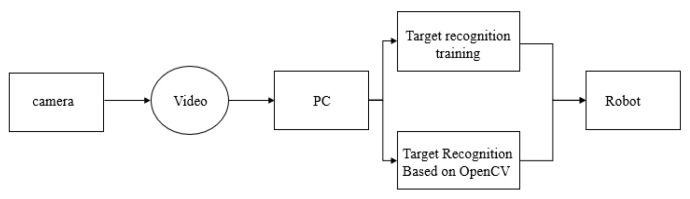
System flow chart.

Computer-based recognition technology refers to technology that uses a computer to process video information shot by a camera so as to recognize the objects in the video. A control flow chart is given in Figure 9 [13].

1. For target object information acquisition and training, the XLM file is recognized by collecting the target object image in the early stage and then using the Haar cascade classifier to detect the target object for recognition training.
2. For information collection around the robot, through the front camera of the robot, the surrounding situation of the car body is monitored, and video images are collected in real time.
3. For target recognition, through the computer, the image information collected by the camera in real time and the target file generated by the training can be processed, which can identify the target object when the robot is running.

### 3.2. Research on Target Recognition System Based on OpenCV

#### 3.2.1. Overview of Cascade Classifier Algorithm

**Figure 10:**
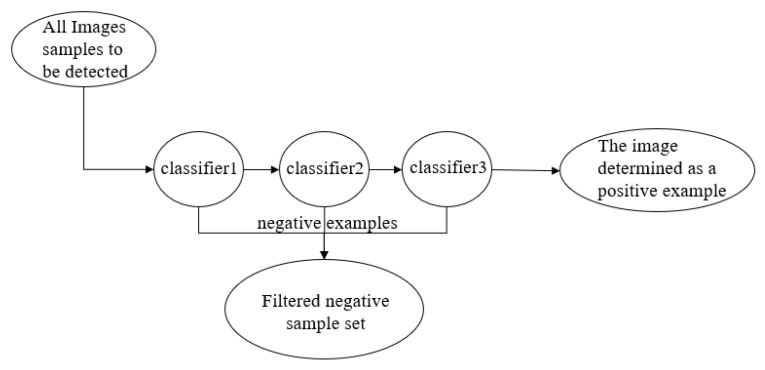
Flow chart of cascade classification.

The Haar cascade classifier is mainly used for the location of human facial information. Strong classifiers are cascaded together with a screening cascade, which can carry out more accurate detection [14]. Haar classification is actually an application of the Boosting algorithm, and the AdaBoost algorithm of the Boosting algorithm is used in the Haar classifier. Only the strong classifiers in the AdaBoost algorithm are cascaded, and the efficient integral image and rectangle feature method are used in the underlying feature extraction. In short: Haar classifier = Haar-like features + integral graph method + Adaboost algorithm + cascade [15]. The Haar cascade algorithm implements image training in four main stages: determining Haar-like features, obtaining integral images, AdaBoost training, and classification using a cascade classifier [16]. The cascade classifier model diagram is shown in Figure 10.

#### 3.2.2. Feature Extraction Based on AdaBoost

The Adaboost algorithm combines many weak classifiers with general classification ability into a strong classifier with strong classification ability, and then connects several strong classifiers in series to complete the detection of objects in the image. The requirements of the detection system for error rate and recognition rate are the important basis for the cascade series.

1. Definition of weak classifier
  $$h_{j}\;\left(x\right)=\left\{\begin{array}{cc} 1 & p_{i}\;f_{j}\;\left(x\right)<p_{i}\;\theta_{j}\\ 0 & \mathrm{Other} \end{array}\right.$$
  $h_{j}$ (X) is the classifier value of a Haar-like feature; X is the sub-window to be tested;$\;f_{j}\;\left(x\right)$ is the characteristic value; $\theta_{j}$ is the threshold value; $p_{i}$ is the sign factor.
2. Train a strong classifier

A plurality of weak classifiers constitute a strong classifier, and the strong classifier can better process the object. By cascading the trained strong classifiers, the detection speed of the target object in the image can be accelerated, and the non-target object can be excluded. The strong classifier training process is as follows:

(1) For the known N training samples ($x_{1}$, $y_{2}$), ($x_{2}$ $y_{2}$),…, ($x_{n}$ $y_{n}$), M of the N samples are non-target samples, and L are target samples (Matryoshka). Sample with = 0$w_{1\sim M}$ = $1/\left(2M\right)$, and for $y_{i}$, sample with = 1$w_{1\sim L}$ $1/\left(2L\right)$.

(2) Iterate for t times.

① Normalized weight:

$$q_{t,i}\;\frac{w_{t,i}}{\sum_{j=1}^{n}w_{t,j}}$$

② The weighted error rate of the weak classifier $h_{j}$(X) corresponding to each Haar-like feature is calculated:

$$\varepsilon_{j}=\sum_{i}q_{i}\left|h_{j}\left(x_{i}\right)-y_{i}\right|$$

③ Select the weak classifier with the minimum error rate, and $h_{j}$(X) is added to the strong classifier:

$$\varepsilon_{t}{\;\min}_{f,p,\theta }\sum_{i}q_{i}\left|h_{j}\left(x_{i}\right)-y_{i}\right|$$

④ Increase the weight of the weak classifier with the error rate, and decrease the weight of the weak classifier with a small error rate:

$$w_{t+1}\;w_{t,i}\beta_{t}^{1-e_{i}}$$

where *e* = 0 if the input feature is correctly classified; otherwise, *e* = 1.

⑤ After t iterations, the weak classifier is cascaded into a strong classifier:

$$\begin{array}{l} R\left(X\right)=f\left(x\right)=\left\{\begin{array}{cc} 1 & \overset{T}{\underset{t=1}{\sum }}\alpha_{t}h\left(x\right)\geq \frac{1}{2}\overset{T}{\underset{t}{\sum }}\alpha_{t}\\ 0 & \;\;\;\;\;\;\mathrm{Other} \end{array}\right.\\ \alpha_{t}=\;\log\frac{1}{\beta_{t}}\; \end{array}$$

Through the above training, the correct classification of the known samples can be realized, and the training of the wrong samples is strengthened by re-dividing the weight of the samples for T times. Finally, all the weak classifiers are combined by the weight to form a strong classifier.

#### 3.2.3. Target Object Image Acquisition and Recognition Training

**Figure 11:**
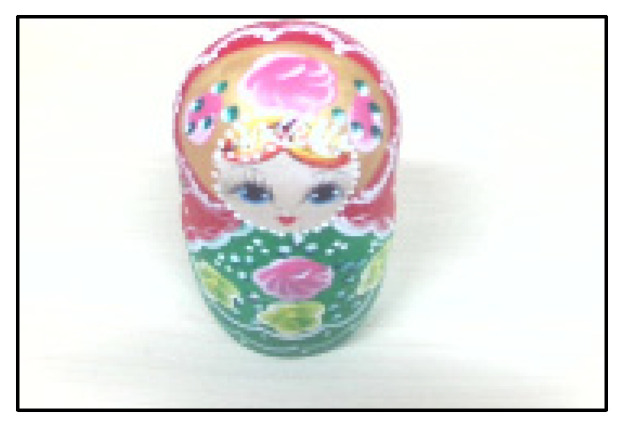
Target object.

Target recognition based on OpenCv needs to collect a large number of images of target samples in the early stage and then train them through OpenCv to obtain the training results, so that the computer can recognize the target object through the camera. The target sample for this study is Matryoshka dolls, as shown in Figure 11.

The recognition system in this study uses a high-definition camera with a focal length of 6 mm and a definition of 720p, which can take real-time high-definition pictures of the surrounding environment of the robot.

**Figure 12:**
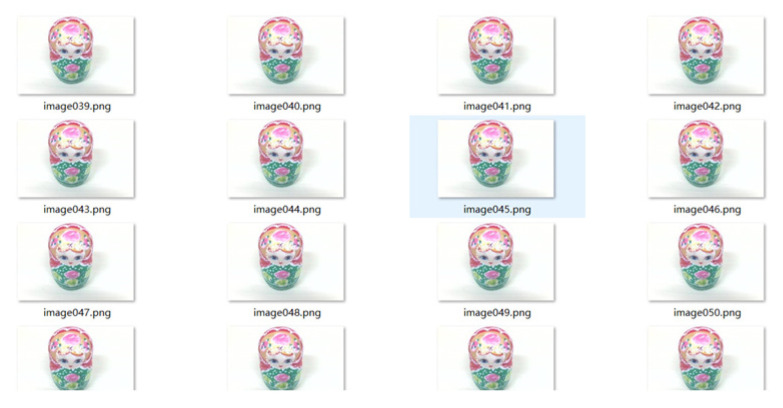
Identify the training process.

The camera records the video and saves each clear image in the video image through OpenCv as the collected target sample image. In this training, 100 images of nesting dolls from all angles are selected as the positive samples, as shown on the right side of Figure 12, and 174 negative sample images are collected. There is no intersection between the negative sample images and the positive sample images. After obtaining the image set of the target detection object, the acquired image is trained by the Haar cascade classifier in OpenCv, and finally, the trained target XLM file is obtained, and the recognition and selection of the target object can be realized through the call of this file in Opencv.

## 4. Educational Experimental Protocol Design

### 4.1. Closed-Loop Servo PID Regulation Experiment

#### 4.1.1. Introduction

**Figure 13:**
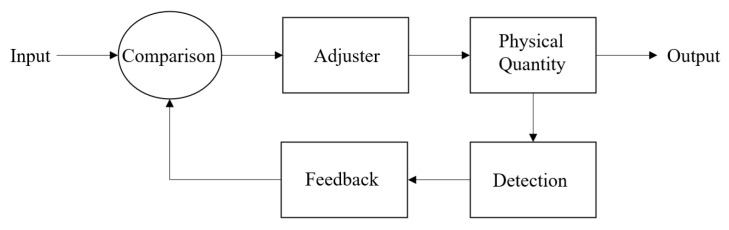
Closed-loop servo program block diagram.

As shown in Figure 13, the closed-loop servo system is an automatic control system, including power amplification and feedback, so that the value of the output variable corresponds to the value of the input variable. This system automatically detects the actual displacement of the worktable and compares it with the command value, controlling it with the difference.

Proportional integral derivative (PID) control is one of the earliest developed control methods. It has the characteristics of a simple algorithm, high reliability, and strong robustness, and it has been widely used in the field of control [17]. Its main purpose is that when the output of the control system deviates from the set value, the PID regulator can quickly and accurately eliminate the deviation and return to the set value.

$$u\;=K_{p}e+\frac{1}{\mathrm{Ti}}\int_{0}^{t}edt+\mathrm{Td}\;\frac{de}{dt}\;$$

#### 4.1.2. Experimental Steps

1. Before adjustment

**Figure 14:**
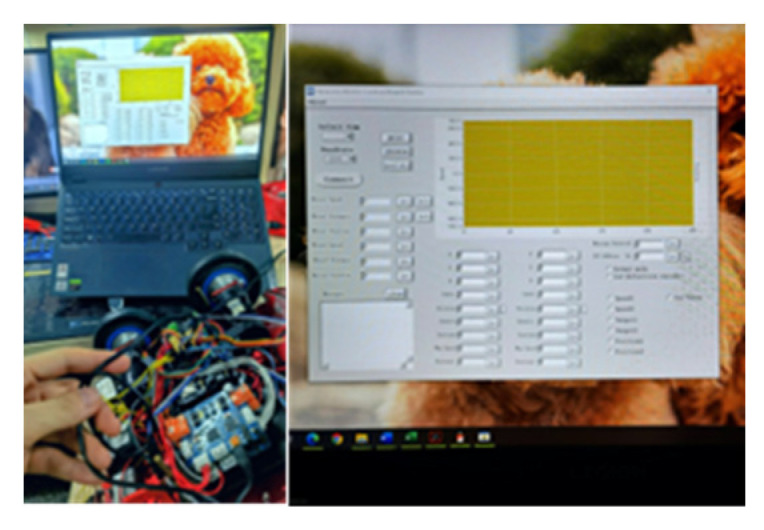
Connection and debugging.

After connecting the motor with the motor drive board, connect the motor drive board to the computer through the data line, as shown in Figure 14 above, provide an independent power supply for the motor, and then adjust the speed of the motor in real time PID through the software Neurons Motor Control Board Demo, and the encoder on the motor feeds back the speed curve of the motor to the computer.

**Figure 15:**
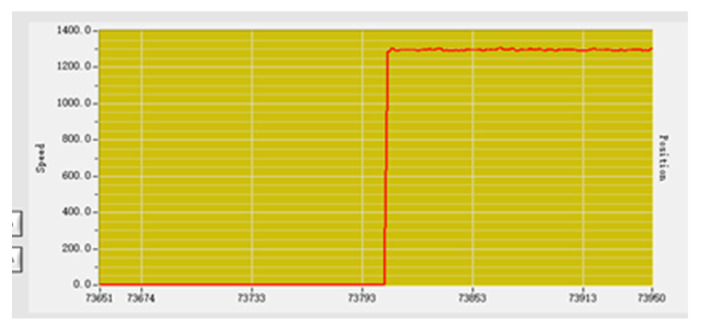
Adjust the previous speed curve.

Before adjustment, the input speed parameter is set to 1000, and the output parameter fluctuates around 1300 after stabilization, as shown in Figure 15. There is an error between the actual output and the intended output.

2. P (Proportion) adjustment

**Figure 16:**
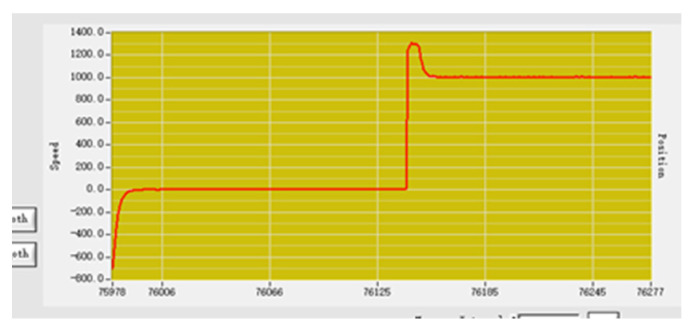
Proportional adjustment speed curve.

P adjustment is carried out with the purpose of eliminating and reducing the error through the proportional adjustment link. Set the P parameter to 100, the speed input is 1000, and the actual speed output is stable at about 1000, as shown in Figure 16, which is basically in line with the expected output [18].

3. PI (Proportional Integral) adjustment

**Figure 17:**
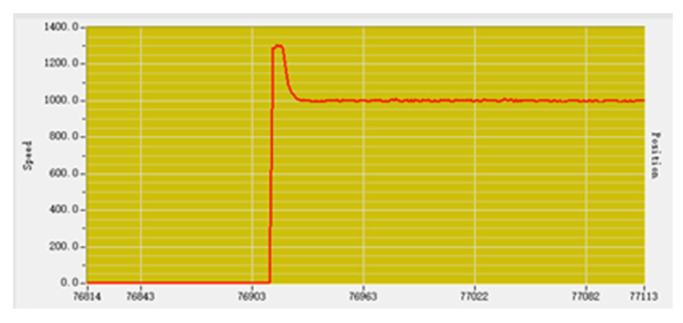
Proportional integration adjusts the velocity curve.

Carry out PI proportional integral regulation and increase integral regulation link to reduce vibration. Set the P value as 100, the I value as 50, and the speed input as 1000. The actual output speed curve is shown in Figure 17.

4. PID (Proportional Integral Derivative) adjustment

**Figure 18:**
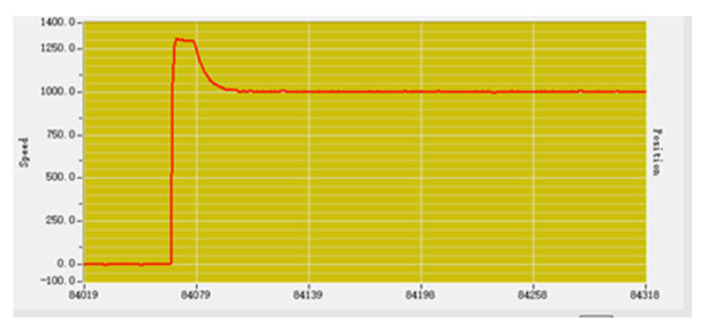
Proportional integral differential adjustment velocity curve.

The D (differential) regulation link is added to the PID proportional integral differential regulation, so as to shorten the regulation time by adding the differential link. Set the P parameter as 100, the I parameter as 50, the D parameter as 20, and the speed input as 1000. The actual output speed curve is shown in Figure 18, which is basically in line with the expected speed output.

#### 4.1.3. Summary

Through proportional adjustment, accelerate the adjustment to reduce the error, and increase the integration link to eliminate the steady-state error of the system and improve the error. The addition of the differential link reflects the change rate of the system deviation signal, which can predict the trend of deviation change and produce advanced control, so the expected desired output characteristics can be obtained after PID adjustment [19].

Through this experiment, students can adjust the speed of the motor by themselves, which can help them to better understand the role and significance of closed-loop servo PID adjustment.

### 4.2. Robot Obstacle Avoidance and Handling Experiments

#### 4.2.1. Introduction

**Figure 19:**
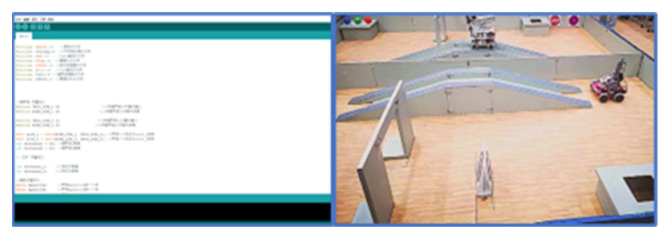
Experimental pictures.

The teaching robot is mainly controlled by the Arduino controller, which is a convenient, flexible, easy-to-use open-source electronic prototype platform. This robot is equipped with a Mega2560 development board. Through the programming of the development board, it can achieve the functions of each part of the car body, The test site and procedure are shown in Figure 19.

#### 4.2.2. Experimental Steps

Students program the development board through Arduino IDE and the IDE window in the figure below. They can use the development board to control the motor drive board to make the steering and speed of the four wheels of the car body achieve different controls and to realize the free movement of the car body through the ultrasonic and infrared sensors installed around it, which are defined in the program and used to detect the situation around the car body. When the ultrasonic feedback distance is less than a certain value, the car body brakes or steers. When the car body gets near to the target grab, it stops and realizes the telescopic grab of the robotic arm through the control of the servo on the robotic arm [20].

#### 4.2.3. Summary

Through this lab, students will be able to experiment with simple programming, and they will learn to use programming languages to control robots, as well as how to use ultrasonic infrared sensors. By defining infrared and ultrasound in the program, the sensor can be called to measure ranging and avoid obstacles, control the robot, and realize the functions of obstacle avoidance and grasping in teaching experiments.

### 4.3. Object Recognition Experiments

**Figure 20:**
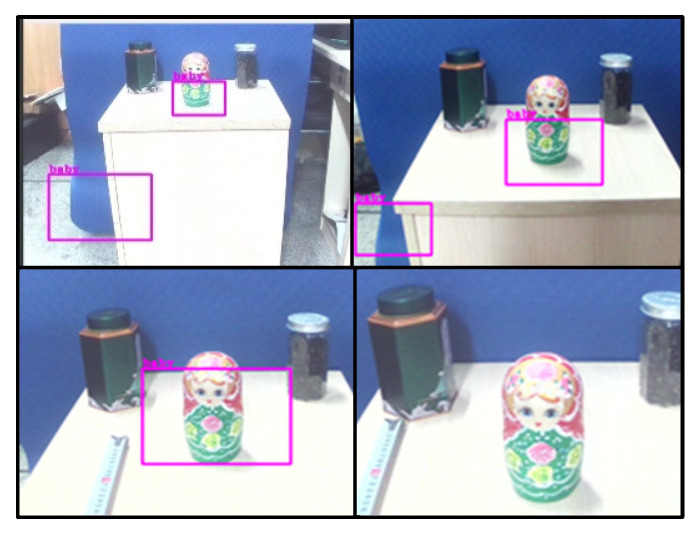
Image of recognition situation.

The identification file of the target object is obtained through the training of the above Haar cascade classifier, and the above identification file is loaded into the program through Pycharm and OpenCv. After the identification program is run, the target object is identified and locked by the camera, and the mark box is shown in Figure 20.

By continuously adjusting the distance between the camera and the recognized object, the statistical results of the distance of the object recognized by the camera are recorded as shown in the Table 2:

Through the target recognition experiment, students can select different target objects for recognition training through cascade classifiers. After obtaining the training files, Opencv can use the camera to complete the recognition and positioning of target objects by robots, cultivate students’ learning of cascade classifiers and Opencv knowledge, and understand image recognition technology.

## 5. Discussion

At present, the research field of teaching robots and their education and teaching system has a long history. Countries that are well versed in science, technology, and education, such as the United States, Germany, Japan, and the United Kingdom, have carried out leading research in this field and made important research progress. These research results not only show the importance of this study, but also its necessity and feasibility. Through the development of educational robots and robot courses, experimental equipment, experimental instructions, competition activities, teacher training, practical training platforms, etc. are effectively integrated to establish a complete professional robot teaching system. Students can develop a step-by-step knowledge improvement process, avoid wasting learning time and teaching resources, and connect textbook knowledge with practice. High-level robot research and teaching courses can be formed in universities.

## 6. Conclusions

The design of this teaching robot delivers a robot designed from aspects of the difficulty of robot construction and software control, equipped with ultrasonic and infrared elements, which can determine its surrounding situation. Through PID control, the movement of the robot can be accurately controlled, so that students can understand the robot servo control system and develop a stronger interest in the application of programming through hands-on application.

This article has accomplished the following: (1) The motion analysis of differential steering of a wheeled robot is achieved. (2) A complete and clear design of a wheeled robot chassis from the motor, driver, and main control board to the sensor is developed. (3) Based on the cascade classifier, an educational robot recognition system is designed, which can accurately identify objects with complex shapes and colors at different distances and different positions; (4) Three course experiments from shallow to deep are designed to help educational robots to be better used in education and teaching.

Considering the many problems existing in the use of educational robots, this paper utilizes knowledge of deep learning, gives a lot of thought to hardware and experimental design, and designs a wheeled educational robot, which improves students’ relevant hardware knowledge and hands-on practical learning, giving them a deeper understanding of cutting-edge knowledge. The application of this educational robot can establish a complete robot education system and improve the quality of courses in the field of domestic robot teaching.

## Figures and Tables

**Table 1:** Symbol meaning.

Letter	Meaning
$v_{x}$	Longitudinal velocity of the center of mass
$v_{y}$	Transverse velocity of the center of mass
α	Angle of sideslip
r	Wheel radius
$w_{l}$	Speed of the left wheel
$w_{r}$	Speed of the right wheel
$\dot{\varphi }$	Yaw rate in the xoy plane about the Z axis
b	Distance between the center of mass of the left and right wheels
$s_{l}$	Slip rate of the left wheel
$s_{r}$	Slip rate of the right wheel

**Table 2:** Identification distance and condition.

Distance (mm)	Identify the Picture	Identify the Situation
120	Top left	Target object can be detected.
80	Top right	Target can be more accurately identified.
40	Bottom left	The recognition situation is the best.
20	Bottom right	The recognition condition flashes.

## Data Availability

The data presented in this study are available upon request from the corresponding author.
